# Complete Genome Sequence of Linezolid-Susceptible *Staphylococcus haemolyticus* Sh29/312/L2, a Clonal Derivative of a Linezolid-Resistant Clinical Strain

**DOI:** 10.1128/genomeA.00494-15

**Published:** 2015-05-21

**Authors:** Lara M. de Almeida, Carlos Pires, Louise T. Cerdeira, Théo G. M. de Oliveira, John Anthony McCulloch, Paula Juliana Perez-Chaparro, Andrey G. Sacramento, Artemir C. Brito, Joás L. da Silva, Maria Rita E. de Araújo, Nilton Lincopan, Melissa J. Martin, Michael S. Gilmore, Elsa M. Mamizuka

**Affiliations:** a Department of Clinical and Toxicological Analyses, Faculty of Pharmaceutical Sciences, University of São Paulo, São Paulo, Brazil; b Centro de facilidades de apoio a pesquisa CEFAP, University of São Paulo, São Paulo, Brazil; c Laboratory of Genetic and Molecular Cardiology, Heart Institute, Faculty of Medicine, University of São Paulo, São Paulo, Brazil; d Faculty of Pharmaceutical Sciences, São Paulo State University, Araraquara, Brazil; e Hospital Beneficência, Portuguesa, São Paulo, Brazil; f Institute of Biomedical Sciences, University of São Paulo, São Paulo, Brazil; g Departments of Ophthalmology, Microbiology and Immunobiology, Harvard Medical School, Massachusetts Eye and Ear Infirmary, Boston, Massachusetts, USA

## Abstract

We report the whole-genome sequence (WGS) of an *in vitro* susceptible derivative revertant mutant from a bloodstream isolate involved in a nosocomial outbreak in Brazil. The WGS comprises 2.5 Mb with 2,500 protein-coding sequences, 16rRNA genes, and 60 tRNA genes.

## GENOME ANNOUNCEMENT

*Staphylococcus haemolyticus*, a commensal coagulase-negative staphylococcus (CoNS) species from the human skin microbiota, has also been increasingly associated with nosocomial infections and resistance to multiple antimicrobials (1, 2). We report the whole-genome sequence of *S. haemolyticus* strain Sh29/312/L2, which is an *in vitro* susceptible derivative from a bloodstream isolate of a linezolid-resistant *S. haemolyticus* clinical strain, Sh29/312. Sh29/312/L2 was isolated on day 7 from serial passages on antibiotic-free Mueller Hinton agar when the linezolid minimum inhibitory concentration (MIC) of Sh29/312 decreased from 64 to 1 µg/mL.

Genome sequencing was performed using the Ion Torrent PGM platform (Life Technologies) and 400-bp kit. The sequences were *de novo* assembled using Mira version 4.0.2 and SPAdes version 3.1.1. The scaffold was obtained using CLC Genomics Workbench version 7.5 and Geneious version 8.0.5 with the genome reference *S. haemolyticus* strain JCSC1435 (GenBank accession number AP006716.1) (3). The genome was annotated with Prokka (4) and curated using Artemis software (5).

The sequence analysis showed that *S. haemolyticus* strain Sh29/312/L2 has 2,561.368 bp with 2,417 protein-coding sequences, 16 rRNA genes, 60 tRNA genes, 39 pseudogenes, and 32.72% GC content. Sh29/312/L2 also harbors one intact and other incomplete phages.

The sequence of the *S. haemolyticus* Sh29/312/L2 genome will provide a reference for a comparative genome analysis with its ancestral linezolid-resistant *S. haemolyticus* clinical strain, which has some resistance mechanism other than those known target-site modifications that promote elevated MIC for linezolid. This research will help provide insight to the genomic background of an important CoNS species. After the important work of Takeuchi and colleagues (3), no whole-genome sequence of *S. haemolyticus* has yet been reported.

### Nucleotide sequence accession number.

The whole-genome sequence of *S. haemolyticus* strain Sh29/312/L2 has been deposited in DDBJ/ENA/GenBank under the accession number CP011116.
